# Image file formats

**DOI:** 10.2349/biij.2.1.e6

**Published:** 2006-01-01

**Authors:** LK Tan

**Affiliations:** Department of Biomedical Imaging, Faculty of Medicine, University of Malaya, Kuala Lumpur, Malaysia

## INTRODUCTION

This tutorial is about image file formats: what are they, what are they used for, what are their differences and how we choose between them. The tutorial assumes a basic understanding of general digital imaging; of which a quick summary of important features is provided below.

*Pixel*: a digital image is represented as a rectangular grid of dots, where each dot has a specific spatial position and is colour defined. Each of these dots is known as pixels, and represents the smallest unit in a digital image.

*Matrix Size*: the dimensions of a digital image; usually specified in terms of its width and height in pixels. For an image with square pixels, the matrix size also works to define the image aspect ratio.

*Spatial Resolution*: a measure of the amount of spatial detail in a digital image. Resolution is stated as a ratio of physical to sampled dimensions, usually in the form of pixels per cm or dots per inch. The stated physical dimensions depend on whether the application is for acquisition or output.

*Colour Space*: colour space or colour specification system is the method used to represent colour. Greyscale or monochrome images usually use a single intensity gradient. Colour images however, often encode colour in multiple channels, combining them to represent an individual colour. Common examples include Red-Green-Blue (RGB) and Cyan-Magenta-Yellow-Black (CMYK).

*Bit Depth*: bit or colour depth may be thought of as colour resolution, i.e., a measure of the amount of colour detail in a digital image. Bit depth states the number of computer storage units (bits) used to represent the colour of each individual pixel in an image. Common values are 24 bits per pixel (bpp) for RGB colour images (8 bits per channel), and 8 bpp for greyscale images. Medical greyscale images often exceed 8 bpp, with 10 bpp and 12 bpp being the most common.

## WHAT IS AN IMAGE FILE FORMAT?

At its most basic level, computers store and work on digital values of zeros and ones, known as bits. These bits of data are then used to represent meaningful information, depending on the context. For example, the bit sequence 01000001 might represent the number 65 in a calculator program, while the same sequence might represent the letter ‘A’ in a word processor program, or the colour ‘dark grey’ in a graphics program.

As eluded to above, without contextual information, data in a computer becomes meaningless. Information about the data (known as metadata) is just as important as the data itself.

An image file format is a standardised specification to encode information about an image into bits of data for storage. In a nutshell, an image saved and encoded to a known image format identifies itself as an image, and provides useful information such as its matrix size and bit depth, to ease interaction with the file. Any program which adheres to the format standard may then open the file and display the image.

## BASIC TYPES OF IMAGE FORMATS

Most people categorise images by their visual content. For example, x-ray films are medical images, photos of hills and valleys are landscape images, paintings are artistic images and so on. In contrast, computers are largely unaware of such visual contextual information, instead categorizing images by their method of representation. The two fundamental image format types are raster images and vector images (some formats however, allow a mix of the two).

### Raster

A raster image, or bitmap, is the more commonly encountered representation form. A bitmap represents an image via a rectangular grid of pixels, where each individual pixel’s spatial location and colour is defined.

**Figure 1:**
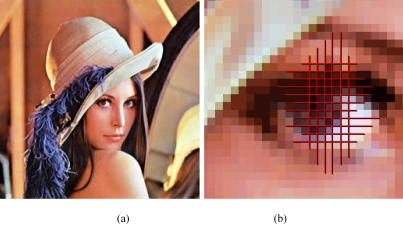
(a) A typical bitmap image. When viewed from a distance, the image appears smooth and continuous; (b) the eye region zoomed in by 10x. Individual pixels – the atomic units that make up the image – are revealed.

Raster images may be thought of as analogous to the human eye, which ‘sees’ via a large cluster of light sensitive cells in the retina. Like a pixel, each cell has a specific spatial location and measures the frequency (colour) and intensity (brightness) of the light at that spot (Figure 1).

The vast majority of medical images are in raster format, which is the focus of this tutorial. Examples of raster formats are BMP, GIF, JPEG, and TIFF.

### Vector

**Figure 2:**
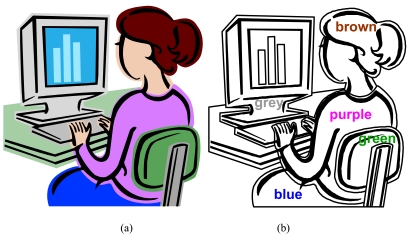
(a) Clean lines and shapes are characteristic of a typical vector image; (b) the image broken down into its primitives, consisting of geometric shapes and lines. Line thickness, fills and colour are specified.

A vector image, or geometric image, represents an image mathematically via the use of geometrical primitives such as points, lines, curves and polygons (Figure 2). In a sense, one may consider a vector image as storing information regarding the shapes in an image rather than the raw image itself. Interestingly, the brain appears to represent images in the same way: we regularly recognize an image by identifying individual objects in the image by pattern and shape.

**Figure 3:**
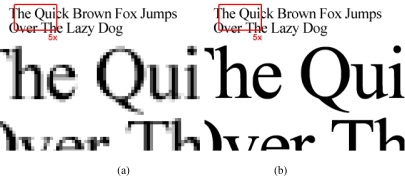
A sentence of text rendered as a bitmap (a) and vector (b) image. When zoomed in by 5x, the bitmap image appears fuzzy and blurry as the resolution has dropped. In contrast, the vector text remains sharp throughout.

An example of a vector image we commonly use is a textual font like Arial or Helvetica. Although most of us would not think of a font in the sense of an ‘image’, a font fulfils all the criteria of a vector image: each character is described by a series of geometric curves and lines. Fonts also demonstrate one of the biggest advantages of vector images: it is scale independent i.e., a font can be scaled to any size without loss in sharpness or detail (Figure 3).

Vector images are not common in the medical field, and will not be covered in this tutorial. An example of their usage is the graphical treatment plan in radiotherapy. Examples of vector formats are WMF, AI, EPS, and SVG.

## IMAGE PARAMETERS

As mentioned previously, an image file stores the raw image pixel data and the metadata, or information about the image. Exactly what metadata to store, and how to do so, depend on the format used. At a minimum, most formats store information regarding the matrix size, colour space, and bit depth. Other common metadata include:

*Compression type*: to save storage space, most formats allow for data compression to be applied on the image data. The two fundamental types of data compression are lossless compression and lossy compression, which will be elaborated further in the following section.

**Figure 4:**
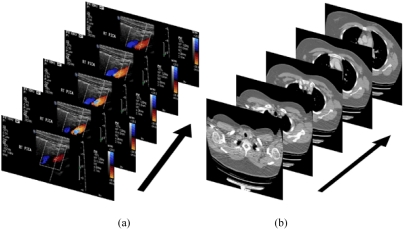
(a) Ultrasound clips use the 3rd dimension to represent time; (b) CT volumetric images use the 3rd dimension to represent depth.

*Dimensions*: certain formats allow for multiple images to be stored in the same file. The motive for doing so however, varies from format to format. For example, formats which support animation will display each image rapidly in sequence as individual animation frames (Ultrasound clip). Other formats, which support 3D data, may use each image as an individual section of the entire volumetric set (CT) (Figure 4).

**Figure 5:**
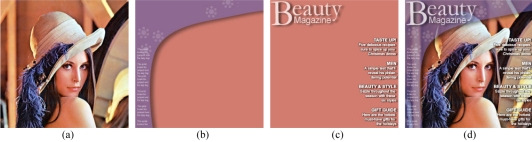
(a, b, c) Separate image layers; (d) a composite of the three layers on the left, where individual layers can “switched” on and off at will.

*Layers*: layers are somewhat similar to dimensions in which they allow for multiple images to be stored in the same file. The difference is that layers are meant to be merged and viewed as a single image, but with the ability to hide individual layers at will. This is useful for applications such as overlays, where the textual information is saved as a separate layer from the actual image of interest (Figure 5).

*Others*: other miscellaneous metadata include date and time of creation, copyright information, comments and others.

The importance of metadata cannot be overstated, as it is vital for proper reconstruction of the image. For example, given a raw image file that is 200 bits in size, there is no way to tell whether the stored image is a 1x2 portrait (10x20 pixels), a 2x1 landscape (20x10 pixels) or indeed anything at all.

## BITMAP IMAGE COMPRESSION

Consider a typical uncompressed CT image – 512 x 512 pixels in size at 12 bits per pixel. Ignoring any extra metadata, the image would need a minimum of 3,145,728 bits or 393,216 bytes of storage space. Multiply the storage needed for a 100 slice study and 10 such studies a week, it is easy to see why digital storage space is a real concern when working with digital imaging. Rather than blindly purchasing hardware to increase the available storage capacity, it makes sense to find ways of utilizing the available storage space more efficiently.

The idea behind image compression is to take advantage of redundancies in the image data, and re-encode the data in such a way to make it more compact. The two fundamental types of data compression are lossless and lossy compression.

### Lossless

**Figure 6:**
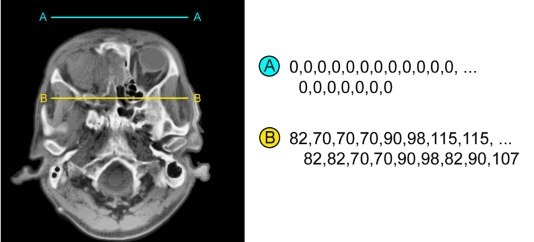
Lossless compression works by taking advantage of patterns and repetitions in data; The long stream of zeros (black) in line profile A–A will easily yield huge compression savings, but the seemingly random profile of B–B will see little benefit.

Lossless compression works to reduce mathematical redundancy. This means the algorithm searches for repeating patterns or sequences in the images, and reduces them to a compact formula. For example, in a typical CT image, the surrounding edges are usually totally black (air), which would be represented by a long sequence of zeros (0 0 0 0 0 0 0 0 0 0 …). The lossless algorithm would detect the sequence and replace it with an encoded formula of the form ‘repeat zero ten times’ (Figure 6).

When the compressed image is decompressed for viewing, the resulting image is identical to the original source image, i.e., no information was lost in the compression process. Lossless formats typically achieve 1:2 space savings or so, and work best with relatively simple images such as diagrams and line art; images with clean lines and flat colour.

### Lossy

Lossy compression works to reduce perceptual redundancy. This means the algorithm takes into account limitations of the human eye, and discards data that is deemed nonessential to the perceptual quality of the overall image. For example, the human eye is less sensitive to colour details as well as very bright and very dark tones. The lossy algorithm might then reduce the spatial resolution of the colour channels, and smooth the parts of the image that are very bright and very dark.

**Figure 7:**
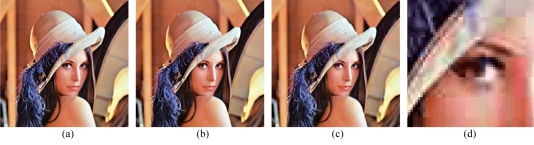
(a, b, c) Lossy compression artifacts: the sample image is saved at progressively higher compression levels, starting at 1:1, then 1:10, then 1:30. Even at 1:30, the image remains very legible, though obvious visual glitches are present; (d) the 1:30 compressed image when zoomed at 5x. There is obvious smudging of detail, as well as a “blocking” artifact; a key characteristic of the JPEG lossy compression format.

It should be emphasised that unlike lossless compression, the final decoded lossy image is not identical to the original source. The more aggressive the compression, the more information is discarded and the more noticeable the difference between the compressed image and the original. For this reason however, lossy compression typically achieves better results, at around 1:10 space savings or so. Lossy compression works best with photographic images, or images comprised of gradients and tones with few sharp edges (Figure 7).

## COMMON BITMAP IMAGE FILE FORMATS

### BMP – Windows Bitmap Format

BMP was introduced by Microsoft® to be the native bitmap image format for their Microsoft Windows® environment. Being a relatively simple format, BMP lacks many features of other, more robust formats, but is supported by most applications and is often thought of as the lowest common denominator format for interchanging images between programs.

### JPEG – Joint Photographic Experts Group

Developed around 1986, JPEG is the *de facto* standard for lossy image compression. In common usage however, JPEG is often used to refer to the JFIF (JPEG File Interchange Format) image format, which utilises JPEG compression. Thus, when someone refers to a JPEG file, she actually means a JFIF file; correspondingly, it is possible for other image formats to utilize JPEG compression as well.

### TIFF – Tagged Image File Format

TIFF was designed to be a highly extensible file format: capabilities could be added to any file by the use of ‘tags’, hence the name ‘tagged image file format’. This resulted in TIFF becoming one of the most featured rich formats, but conversely resulting in many incompatibility issues, where only a subset of applications supported the more complex or esoteric features such as multi-page encoding or lossy compression.

### DICOM – Digital Imaging and Communications in Medicine

In contrast to the aforementioned general purpose image formats, DICOM was designed specifically for use in the medical industry, defining a specific file format and a set of communication protocols. DICOM shares similarities to TIFF in its ability for extension via the use of custom tags. Unlike TIFF however, most extensions revolved around additional information associated to the image (e.g., modality name, patient birth date and physician in charge) rather than additional features.

## RECOMMENDATIONS

There are two main applications for saving images: storing the original source images and making secondary copies.

All devices which generate digital images have an in-built native image file format. For most medical modalities such as CT and MRI, this would usually be DICOM, whereas digital cameras and the like might use JPEG. For archiving, source images should always be left in their original format.

Transcoding from one format to another always carries the risk of information loss. It might be tempting to convert a DICOM file to TIFF or PNG to take advantage of the better lossless compression performance, but the conversion would strip out unsupported metadata such as patient information. Transcoding from a lossless format to a lossy format carries an even greater danger, as the quantizing nature of the lossy algorithm can be unpredictable. Small details in the original image could easily be discarded as imperceptible noise, and the information lost permanently.

For medical imaging, primary diagnosis should only be carried out on the original source images, as they are guaranteed authentic. For many other applications however, perfect reproducibility is not a requirement, emphasis is placed on storage size and convenience instead. Examples of such usage are images in presentations, general purpose review and so on. We group these usages as secondary copies.

**Figure 8:**
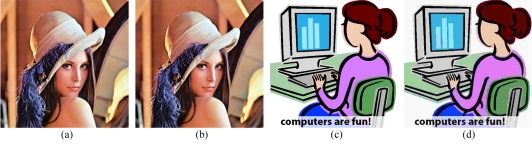
(a) A photo originally in a lossless format (b) when compressed to JPEG, the photo is 10% of its original file size, with little perceptible difference. (c) Simple images compress well in GIF (d) JPEG manages to attain a similar file size, but at the cost of severe image artefacts.

When saving images for secondary copy use, the choice of format is determined primarily on the content of the image. In general, ‘photographic’ images or images with smooth tones and few sharp edges (this includes most medical images) are best compressed with a lossy format such as JPEG. In contrast, images that consist mostly of clean lines and solid colour such as diagrams and text will be best compressed by a lossless format such as TIFF (Figure 8).

**Table 1:** Summary of file formats

	BMP	DICOM	GIF	JPEG	JPEG 2000	PNG	PSD	TIFF
Extension	bmp	dcm	gif	jpeg, jpg, jif, jfif	jp2, jpx, jpc, j2k	png	psd	tif, tiff
Bit Depth (grey)	1, 4, 8	8, 16	1, 4, 8	8	8, 16	1, 4, 8, 16	1, 4, 8, 16	8, 16
Bit Depth (colour)	1, 4, 8, 24	8, 24, 48	1, 4, 8	24	24, 48	1, 4, 8, 24, 48	1, 4, 8, 24, 48	8, 24, 48
Compression*(lossless)(lossy)	«	««	««	««	«««	«««	«««	«««
Comments	Support for compressed BMP is rare	Complex data element structure often introduces minor incompa-tibilities between vendors	For simple diagrams, GIF often compresses better than others	Support for lossless JPEG compression is rare	JPEG 2000 has yet to garner widespread support	Fully open standard designed to replace GIF and TIFF	Proprietary format used in Adobe® Photoshop®	Support for lossy TIFF compression is rare

* The star rating system is based on relative differences of compression performance to other formats, rather than an absolute performance ranking. A single star difference does not indicate a magnitude of improvement, but rather a noticeable improvement in general.

**Table 2:** Free image viewers

	Irfanview^a^	XnView^b^	Osiris^c^	ImageJ^d^
Platforms*	Win32	Win32, Unix, Mac	Win32, Mac	Win32, Unix, Mac
General support:				
BMP	●	●	●	●
GIF	●	●	●	●
JPEG	●	●	●	●
JPEG2000	●	●		
PNG	●	●		●
PSD	●	●		
TIFF	●	●	●	●
DICOM support:				
8-bit	●	●	●	●
10-, 12-, 16-bit			●	●
DICOM Index			●	
Comments	Useful general purpose image viewer	Useful general purpose image viewer	Unstable when handling large numbers of files	Functionality extendible via plug-ins

a Irfanview (www.irfanview.com)

b XnView (www.xnview.com)

c Osiris (www.sim.hcuge.ch/osiris/)

d ImageJ (rsb.info.nih.gov/ij/)

* Win32 encompasses all modern Windows^®^ operating systems from Windows 95 onwards. Unix encompasses all modern Unix compatible operating systems including Linux, BSD and Solaris. Mac encompasses all versions of Mac OS X.

## Notes

Information technology (IT) is currently spurring the rapid advancement in healthcare, especially in biomedical imaging. ***biij*** is launching a new series of tutorial articles called ‘Technology in Imaging Tutorial’ with the objectives to assist researchers and practitioners to keep abreast with current technology and learn how to use it effectively in their research and clinical practice – Ed.
